# Lessons learned from an e-mental health intervention: The promotion of stopblues in 41 french cities

**DOI:** 10.1192/j.eurpsy.2021.318

**Published:** 2021-08-13

**Authors:** K. Turmaine, A. Le Jeannic, A. Dumas, K. Chevreul

**Affiliations:** 1 Eceve Umr1123, Inserm l Université Paris Diderot - Paris 7, Paris, France; 2 Urc Eco, Assistance Publique - Hôpitaux de Paris, Paris, France

**Keywords:** digital health, e-mental health, intervention research, community health

## Abstract

**Introduction:**

For more than a decade, digital health has held promise for enabling a much broader population to have access to health information, education and services. However, the increasing number of studies on the subject show mixed results and currently, there is a certain disillusionment regarding its benefits. And yet, the Covid-19 crisis has revealed the importance of developing digital-based complementary support to existing resources.

**Objectives:**

Factors associated with higher utilization rates among the target audience need to be investigated.

**Methods:**

In 2018, 41 French cities enrolled in an intervention program aimed at promoting StopBlues®, a digital health tool that helps prevent mental distress and suicide among the general population. After two years of experimentation, a Multiple Correspondence Analysis (MCA) was performed using quantitative and qualitative data collection methods from institutional sources, questionnaires and web analytics tools.

**Results:**

Finding trends show that higher utilization rates were associated with the involvement of general practitioners (GPs) in the promotion of StopBlues and the use of digital marketing channels. Context-specific characteristics also played an important role in the adoption of the tool.

**Conclusions:**

The local context has a strong influence on how digital tools are locally promoted and accepted. Further research is needed to understand how local actors and specifically GPs can be involved in suicide prevention. More broadly, the challenge today is to ensure acceptance of digital health technology among targeted populations by adapting the digital offer to their needs and promoting the available tools.

**Disclosure:**

No significant relationships.

